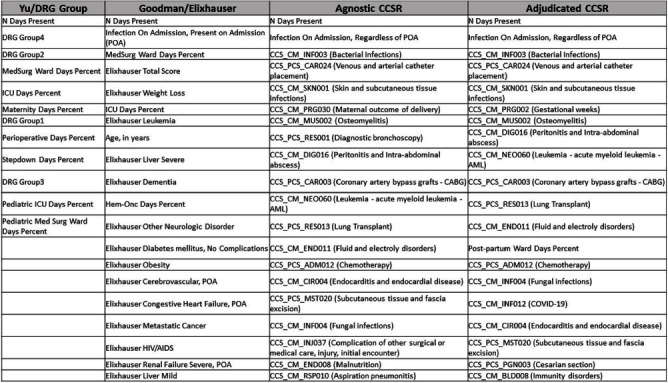# A Comparison of Variable Input Strategies used for Risk-adjustment Models of Antimicrobial Use

**DOI:** 10.1017/ash.2024.139

**Published:** 2024-09-16

**Authors:** Rebekah Moehring, Michael Yarrington, Elizabeth Dodds Ashley, Rachel Addison, Whitney Buckel, Sara Cosgrove, Eili Klein, Carlos Santos, Emily Spivak, William Trick, David J Weber, Congwen Zhao, Deverick Anderson, Benjamin Goldstein

**Affiliations:** Duke University Medical Center; Duke University; Intermountain Healthcare; Johns Hopkins University School of Medicine; Rush University Medical Center; University of Utah School of Medicine and Salt Lake City VA; Cook County Health; University of North Carolina at Chapel Hill; Duke Center for Antimicrobial Stewardship and Infection Prevention

## Abstract

**Background:** External comparisons of antimicrobial use (AU) may be more informative if adjusted for encounter characteristics. Optimal methods to define input variables for encounter-level risk-adjustment models of AU are not established. **Methods:** This retrospective analysis of electronic health record data included 50 US hospitals in 2020-2021. We used NHSN definitions for all antibacterials days of therapy (DOT), including adult and pediatric encounters with at least 1 day present in inpatient locations. We assessed 4 methods to define input variables: 1) diagnosis-related group (DRG) categories by Yu et al., 2) adjudicated Elixhauser comorbidity categories by Goodman et al., 3) all Clinical Classification Software Refined (CCSR) diagnosis and procedure categories, and 4) adjudicated CCSR categories where codes not appropriate for AU risk-adjustment were excluded by expert consensus, requiring review of 867 codes over 4 months to attain consensus. Data were split randomly, stratified by bed size as follows: 1) training dataset including two-thirds of encounters among two-thirds of hospitals; 2) internal testing set including one-third of encounters within training hospitals, and 3) external testing set including the remaining one-third of hospitals. We used a gradient-boosted machine (GBM) tree-based model and two-staged approach to first identify encounters with zero DOT, then estimate DOT among those with >0.5 probability of receiving antibiotics. Accuracy was assessed using mean absolute error (MAE) in testing datasets. Correlation plots compared model estimates and observed DOT among testing datasets. The top 20 most influential variables were defined using modeled variable importance. **Results:** Our datasets included 629,445 training, 314,971 internal testing, and 419,109 external testing encounters. Demographic data included 41% male, 59% non-Hispanic White, 25% non-Hispanic Black, 9% Hispanic, and 5% pediatric encounters. DRG was missing in 29% of encounters. MAE was lower in pediatrics as compared to adults, and lowest for models incorporating CCSR inputs (Figure 1). Performance in internal and external testing was similar, though Goodman/Elixhauser variable strategies were less accurate in external testing and underestimated long DOT outliers (Figure 2). Agnostic and adjudicated CCSR model estimates were highly correlated; their influential variables lists were similar (Figure 3). **Conclusion:** Larger numbers of CCSR diagnosis and procedure inputs improved risk-adjustment model accuracy compared with prior strategies. Variable importance and accuracy were similar for agnostic and adjudicated approaches. However, maintaining adjudications by experts would require significant time and potentially introduce personal bias. If findings are confirmed, the need for expert adjudication of input variables should be reconsidered.

**Disclosure:** Elizabeth Dodds Ashley: Advisor- HealthTrackRx. David J Weber: Consultant on vaccines: Pfizer; DSMB chair: GSK; Consultant on disinfection: BD, GAMA, PDI, Germitec

**Figure:**
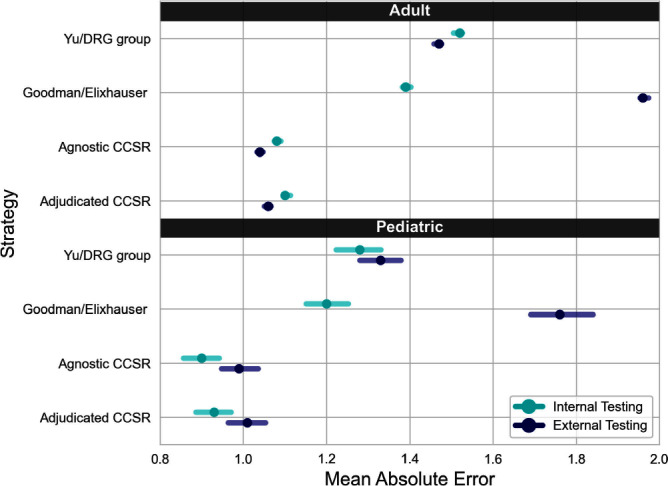

**Figure:**
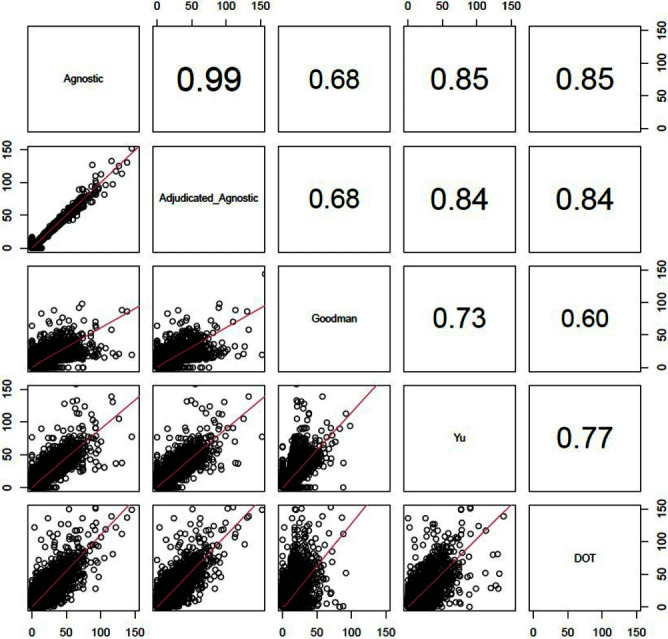

**Figure:**